# Genome-wide identification and expression profiling of *DREB* genes in *Saccharum spontaneum*

**DOI:** 10.1186/s12864-021-07799-5

**Published:** 2021-06-17

**Authors:** Zhen Li, Gang Wang, Xihui Liu, Zhengchao Wang, Muqing Zhang, Jisen Zhang

**Affiliations:** 1 Fujian Provincial Key Laboratory of Haixia Applied Plant Systems Biology, College of Agriculture, Fujian Agriculture and Forestry University, Fuzhou, 350002 China; 2 Jiangsu Key Laboratory for Bioresources of Saline Soils, Jiangsu Provincial Key Laboratory of Coastal Wetland Bioresources and Environmental Protection, Jiangsu Synthetic Innovation Center for Coastal Bio-agriculture, Yancheng Teachers University, Yancheng, 224051 China; 3 Sugarcane Research Institute, Guangxi Academy of Agricultural Sciences, Nanning, 530007 Guangxi China; 4 Provincial Key Laboratory for Developmental Biology and Neurosciences, College of Life Sciences, Fujian Normal University, Fuzhou, 350007 China; 5 Guangxi Key Laboratory of Sugarcane Biology, Guangxi University, Nanning, 530004 Guangxi China

**Keywords:** *Saccharum spontaneum*, DREB, Phylogenetic analysis, Gene expression, Dehydration response

## Abstract

**Background:**

The dehydration-responsive element-binding proteins (DREBs) are important transcription factors that interact with a DRE/CRT (C-repeat) sequence and involve in response to multiple abiotic stresses in plants. Modern sugarcane are hybrids from the cross between *Saccharum spontaneum* and *Saccharum officinarum*, and the high sugar content is considered to the attribution of *S. officinaurm*, while the stress tolerance is attributed to *S. spontaneum*. To understand the molecular and evolutionary characterization and gene functions of the *DREBs* in sugarcane, based on the recent availability of the whole genome information, the present study performed a genome-wide in silico analysis of *DREB* genes and transcriptome analysis in the polyploidy *S. spontaneum*.

**Results:**

Twelve *DREB1* genes and six *DREB2* genes were identified in *S. spontaneum* genome and all proteins contained a conserved AP2/ERF domain. Eleven *SsDREB1* allele genes were assumed to be originated from tandem duplications, and two of them may be derived after the split of *S. spontaneum* and the proximal diploid species sorghum, suggesting tandem duplication contributed to the expansion of *DREB1*-type genes in sugarcane. Phylogenetic analysis revealed that one *DREB2* gene was lost during the evolution of sugarcane. Expression profiling showed different *SsDREB* genes with variable expression levels in the different tissues, indicating seven *SsDREB* genes were likely involved in the development and photosynthesis of *S. spontaneum*. Furthermore, *SsDREB1F*, *SsDREB1L*, *SsDREB2D*, and *SsDREB2F* were up-regulated under drought and cold condition, suggesting that these four genes may be involved in both dehydration and cold response in sugarcane.

**Conclusions:**

These findings demonstrated the important role of *DREBs* not only in the stress response, but also in the development and photosynthesis of *S. spontaneum*.

**Supplementary Information:**

The online version contains supplementary material available at 10.1186/s12864-021-07799-5.

## Background

Plants are exposed to various abiotic stresses such as drought, salinity, and extreme temperature, which cause adverse effects on their growth and yield [1]. A number of genes are induced or repressed by these stresses to help plants to survive from these bad conditions, which can be divided into the gens coding stress tolerance proteins and the other coding regulatory proteins [2, 3]. Transcription factors (TFs) are necessary for regulating the expression of stress-responsive genes. Dehydration responsive element binding proteins (DREBs) are the important TFs that regulate stress-responsive genes expression in the abscisic acid (ABA)-independent pathway [4]. DREBs belong to a subfamily of the APETALA2/ethylene-responsive element-binding protein (AP2/ERFBP) superfamily of TFs, and can bind a dehydration-responsive element (DRE) with the core motif A/GCCGAC that was found in the promoter of many dehydration- and cold stress-inducible genes [1, 5]. Each DREB protein contains a conserved AP2/ERF DNA-binding domain, which consist of ~ 60 amino acids [6, 7]. The three-dimensional structure of AP2/ERF domain revealed this domain comprises a three-strand antiparallel β-sheet and an α-helix packed similarly parallel to the β-sheet [8]. Two amino acids, the 14th valine (V14) and 19th glutamic acid (E19) in the AP2/ERF domain of DREB proteins are conserved and play a central role in determining the DNA-binding specificity of DREB proteins [1]. On the basis of the similarities in the AP2/ERF domain, DREB subfamily has been divided into 6 subgroup (A-1 to A-6), and the canonical DREB proteins belong to subgroups A-1 (DREB1) and A-2 (DREB2) [1, 9].

Though *DREB* genes are mainly involved in the process regulating the drought stress, other functions have been noted for some *DREB* genes. Previous studies have demonstrated that *DREB* genes can be induced by various abiotic stresses, including drought [10–13], low temperatures [14–17], heat stress [18–20] and high salt [21–23]. Overexpressing *OsDREB2A* in soybean enhanced salt tolerance by accumulating osmolytes and improving the expression levels of some stress-responsive genes and TFs [24]. In transgenic *Salvia miltiorrhiza*, *AtDREB1A* and *AtDREB1B* both play a positive role in plant drought stress tolerance [12, 25]. *PvDREB1C* gene is transcriptionally down-regulated in response to salt stress, whereas *PvDREB1C* overexpression improves plant salt tolerance in transgenic tobacco. On the other hand, ectopic overexpression of *PvDREB1C* has been characterized as a negative regulator of cold stress response [16]. *StDREB2* has been reported to play an important role in the drought stress tolerance of cotton (*Gossypium barbadense* L.) [26].

Sugarcane (*Saccharum* spp.) is a major crop mostly grown in tropical and subtropical regions worldwide, and adversely affected by drought, salinity, low temperature, high temperature, etc. Modern sugarcane cultivars are complex autopolyploid and aneuploidy of interspecific hybrids derived mainly from *S. officinarum* and *S. spontaneum*. For *Saccharum* hybrid, *S. officinarum* was assumed to contribute to genetic background of high sugar content, and *S. spontaneum* contributed to the stress tolerance and pest and disease resistance [27]. In China, over 70% of sugarcane were cultivated in the hilly area which contained a low level of soil water content during the drought season. Thus, enhancing drought tolerance has been an important target for improving the yield of sugarcane in field. According to previous researches, transgenic sugarcane transformed with *AtDREB2A CA* showed the enhanced drought tolerance without biomass penalty [28]. Overexpression of *EaDREB2* (*Erianthus arundinaceus DREB2*) in sugarcane enhances the drought and salinity tolerance, what’s more, co-transformation of *EaDREB2* and *PDH45* (pea DNA helicase gene) shows lower drought tolerance but higher salinity tolerance than *EaDREB2* alone [29]. Huang et al. recently had analyzed the DREB subfamily in *S. spontaneum* [30], here, we focused on the canonical *DREB* genes (*DREB1s* and *DREB2s*) and discriminated the genes and their alleles. We also explored the gene function based on large scales of expression profiles from RNA-seq data sets including leaf developmental gradient, diurnal cycle, development stage, drought stress and cold stress. Thus, this study may provide insights into the polyploid characterizes for the *DREBs* and function relative to photosynthesis and plant development beside the drought stress.

## Results

### Identification of *SsDREB* genes in *S. spontaneum* genome

A total of 277 proteins containing AP2/ERF domain(s) were originally obtained in the sugarcane *S. spontaneum* AP85–441 (1n = 4x = 32) genome. Based on the classification of the AP2/ERF superfamily in *Arabidopsis* [1], 54 of them, containing multiple AP2/ERF domains, were classified into the AP2 subfamily. Thirteen of these proteins, possessing both AP2/ERF and B3 domains, were belonged to the RAV subfamily. Thirty-one proteins lacked a conserved WLG motif. Of the remaining 179 proteins, containing only one AP2/ERF domain with a conserved WLG motif, 83 were classified into the DREB subfamily (Group A) and 96 were classified into the ERF subfamily (Group B). Two canonical subgroups of DREBs (DREB1 and DREB2) were 20 and 10 proteins in *S. spontaneum*, respectively. After re-annotating manually with the assistance of FGENESH (http://www.softberry.com/berry.phtml?topic=fgenesh&group=programs&subgroup=gfind) [31], one protein in DREB1 subgroup was identified without AP2/ERF domain and deleted for further researching. Furthermore, these *DREB* genes have 1 to 4 alleles, including 1 gene with four alleles, 2 genes with three alleles, and 4 genes with two alleles (Additional File 1). Based on their chromosomal locations, we renamed these *DREB1s* and *DREB2s* as *SsDREB1A* to *SsDREB1L*, and *SsDREB2A* to *SsDREB2F*, respectively, and additional − 1 to − 4 were added to the gene name for their alleles (Additional File 1).

Gene characteristics, including the length of protein sequences (AA), the molecular weight (MW), the theoretical isoelectric point (pI), the aliphatic index (AI), the grand average of hydropathicity (GRAVY), and the instability index (II) were analyzed (Additional File 1). The protein length were ranged from 186 to 390 aa, while the MW of the proteins from 20,362.44 Da to 41,745.7 Da, and the pI from 4.78 to 10.53 (Additional File 1).

### Multiple sequence alignment and phylogenetic analysis of SsDREBs

All SsDREB protein sequences were found to have an AP2/ERF domain, with a highly conserved WLG motif (Additional File 2). Additionally, SsDREB2 proteins possessed a conserved 14th valine (V14) and a 19th glutamic acid (E19), whereas SsDREB1A to SsDREB1I did not have the glutamic acid in the E19 position (Additional File 2). In DREB1 subgroup, a nuclear localization signal (NLS) sequence ‘P/KKR/KP/RA/TGRT/KKFRETRHP’ and a DSAW motif nestle up to the AP2/ERF domain in the upstream and downstream, respectively. The LWSY motif was found at the end of the C-terminal region in most SsDREB1 proteins, except for SsDREB1A-1, SsDREB1A-3, SsDREB1F-2 and SsDREB1J (Additional File 2). In comparison with DREB1s, all DREB2 protein contained a CMIV-1 ([K/R]GKGGPxN) motif, and a PKK-like NLS sequence ‘RKxPAKGSKKGCMxGKGGPENxx’ was found at the upstream of AP2/ERF domain except SsDREB2E (Additional File 2).

**Fig. 1:**
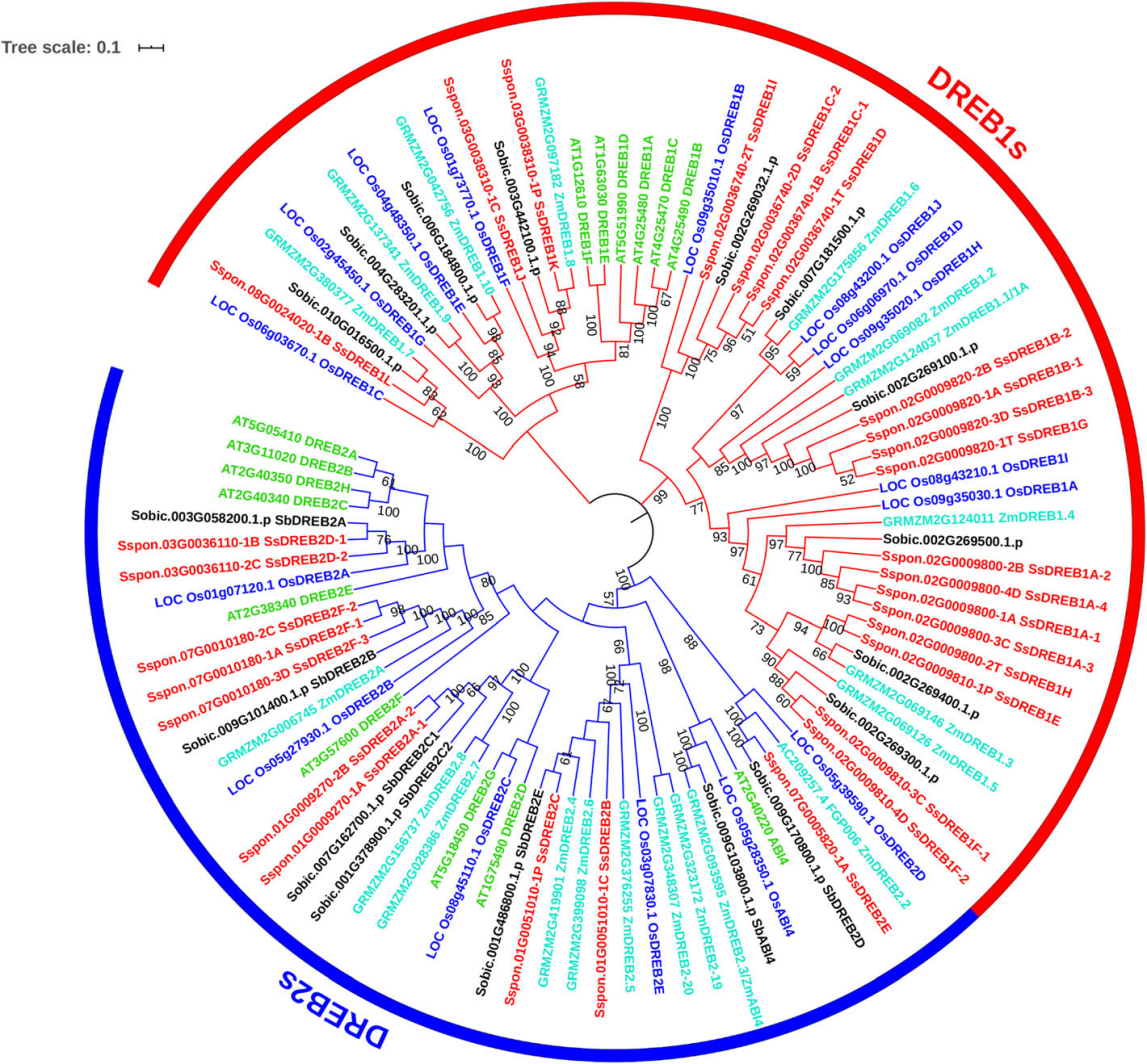
Phylogenetic tree of *DREB1* and *DREB2* genes in *S. spontaneum* (red), sorghum (black), maize (cyan), rice (blue), and *Arabidopsis* (green). The phylogenetic tree was constructed based on the full-length sequence alignments of 97 DREB proteins from five species. Red and blue arcs indicate the *DREB1*-type and *DREB2*-type genes, respectively

In this study, we collected the *DREB* orthologous in *Arabidopsis*, rice, maize and sorghum (Table 1). It’s worth noting that there are two more *DREB1* genes and one less *DREB2* genes in *S. spontaneum* than that in the proximal species sorghum. A phylogenetic tree of the SsDREB proteins and their orthologous was constructed (Fig. 1). Interestingly, the AtDREB proteins were clustered separately from the proteins which were derived from monocots in the *DREB1*-type genes, while clustered together with other proteins in the *DREB2*-type genes. A *DREB2*-type gene *ABI4* belongs to the A-3 subgroup, and those identified in *Arabidopsis*, rice, maize and sorghum were formed a clade, but not found in *S. spontaneum* (Fig. 1), indicating that *ABI4* gene may be lost after the species divergence between *S. spontaneum* and sorghum.

**Table 1:** The number of *DREB* genes in *Arabidopsis*, maize, rice, sorghum, and *S. spontaneum*

Species	DREBs/CBFs		Total
	DREB1	DREB2	
*A.thaliana*	6	9	15
*Z.mays*	10	10	20
*O.sativa*	10	6	16
*S.bicolor*	10	7	17
*S.spontaneum*	12(19)	6(10)	18(29)

The numbers in parenthesis detail the number of alleles of *SsDREBs* in *S. spontaneum*

### Location and duplication events among *SsDREB* genes

**Fig. 2:**
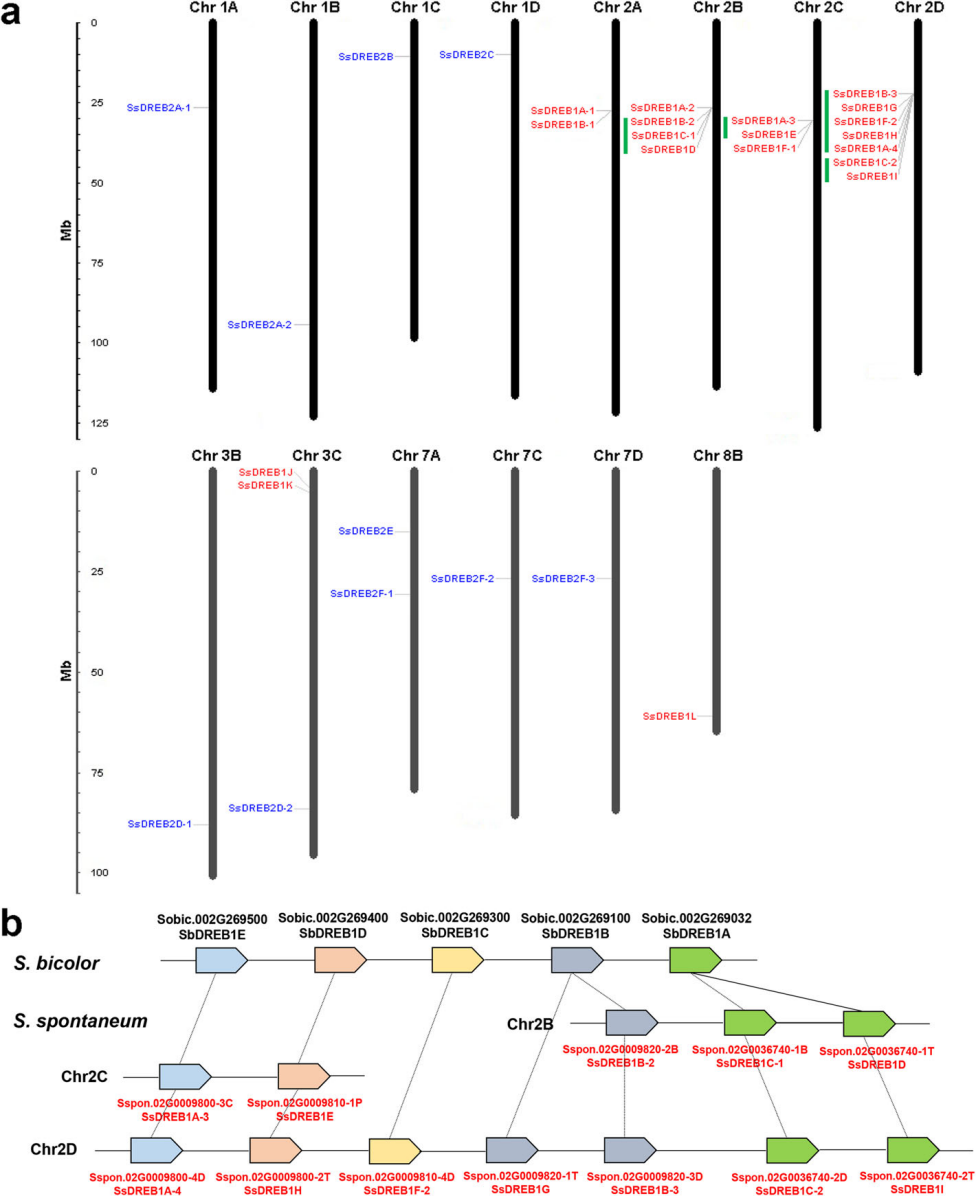
Chromosome distribution of *SsDREB* genes and gene model. **a** The chromosome distribution of *SsDREB* genes. The chromosomal position of *SsDREB* was mapped according to the *S. spontaneum* genome. The chromosome numbers were shown at the top of each chromosome. The scale is in mage bases (Mb). The green lines indicate the tandem duplication regions. **b** Gene model of the tandemly duplicated regions. The colored boxes and lines indicate *DREB* genes and chromosomes, respectively

The genome chromosome location information of *SsDREBs* showed that these 29 *DREB* alleles were unevenly distributed on the 14 chromosomes of *S. spontaneum* (Fig. 2a). Chromosome 2 (2A, 2B, 2C and 2D) contained the largest number of *SsDREB* genes, in addition to chromosome 7A with two *SsDREB2* genes and other chromosomes only with one *SsDREB2* gene (Fig. 2a).

Furthermore, according to the methods of Holub [32], a chromosomal region within 200 kb containing two or more genes is defined as a tandem duplication event. We identified 12 *SsDREB1* allele genes (*SsDREB1B*-2/*1C*-*1*/*1D*, *SsDREB1A*-*3*/*1E*, *SsDREB1B*-*3*/*1G*/*1F*-*2*/*1H*/*1A*-*4*, and *SsDREB1C*-*2*/*1H*), which were clustered into four tandem duplication event regions by BLASTP and MCScanx software, these tandemly duplicated regions were distrusted on the chromosome 2B, 2C and 2D (Table 2). Chromosome 2D had two clusters, indicating a hot spot of *DREB* gene distribution. What’s more, 17 *SsDREB* allele genes were results of the segmental duplication or whole-gnome duplication events, including all *SsDREB2* genes (Additional File 3).

**Table 2:** Tandem duplication events in the *SsDREB* genes

Cluster number	Gene name	Chromosome	Start site	End site
1	SsDREB1C-2	Chr2D	22,208,944	22,209,639
	SsDREB1I	Chr2D	22,226,936	22,227,607
2	SsDREB1B-3	Chr2D	22,092,288	22,093,103
	SsDREB1G	Chr2D	22,107,221	22,108,036
	SsDREB1F-2	Chr2D	22,113,187	22,113,903
	SsDREB1H	Chr2D	22,117,638	22,118,345
	SsDREB1A-4	Chr2D	22,126,308	22,127,015
3	SsDREB1B-2	Chr2B	26,564,597	26,565,409
	SsDREB1C-1	Chr2B	26,597,272	26,598,093
	SsDREB1D	Chr2B	26,612,975	26,613,670
4	SsDREB1A-3	Chr2C	30,543,015	30,543,701
	SsDREB1E	Chr2C	30,554,216	30,554,923

Among these tandemly duplicated gene pairs, *SsDREB1C-2* and *SsDREB1I*, *SsDREB1C-1* and *SsDREB1D*, possessed only one orthologous gene *SbDREB1A*, while the orthologous *SbDREB* genes of *SsDREB1A*-*3*/*1E* and *SsDREB1B*-*3*/*1G*/*1F*-*2*/*1H*/*1A*-*4* were also identified as tandemly duplicated gene pairs (Fig. 2b), indicating that tandem duplication events of *SsDREB1C-2* and *SsDREB1I*, *SsDREB1C-1* and *SsDREB1D* may happened after the divergence between *S. spontaneum* and sorghum. We therefore estimated the divergence time between tandemly distributed *SsDREB* genes and their orthologous *SbDREBs* based on the pairwise Ks (Table 3). The divergence time between *S. spontaneum* and its closest related diploid species sorghum had been estimated by Zhang et al. [33], it is 7.779 million years ago (Mya). In the current study, the divergence time between tandem-duplicated *SsDREB1s* and their orthologous *SbDREB1s* were ranged from 6.487 Mya to 18.874 Mya. In addition, the divergence time of *SsDREB1C*-*2* and *SsDREB1D* with their orthologous were 6.487 Mya and 6.496 Mya, respectively, which are shorter than that of *S. spontaneum* and sorghum (7.779 Mya).

**Table 3:** The divergence time between tandem-duplicated *SsDREB* genes and their orthologous *SbDREBs*

Gene pairs	Ks	Divergence time (Mya)
SbDREB1A-SsDREB1C-2	0.079	6.487
SbDREB1A-SsDREB1D	0.079	6.496
SbDREB1A-SsDREB1I	0.096	7.841
SbDREB1D-SsDREB1E	0.133	10.917
SbDREB1D-SsDREB1H	0.140	11.496
SbDREB1A-SsDREB1C-1	0.170	13.902
SbDREB1B-SsDREB1B-2	0.180	14.747
SbDREB1B-SsDREB1B-1	0.183	15.038
SbDREB1B-SsDREB1G	0.191	15.686
SbDREB1E-SsDREB1A-4	0.213	17.444
SbDREB1E-SsDREB1A-2	0.230	18.874

### Gene structure and motif composition analysis of *SsDREBs*

**Fig. 3:**
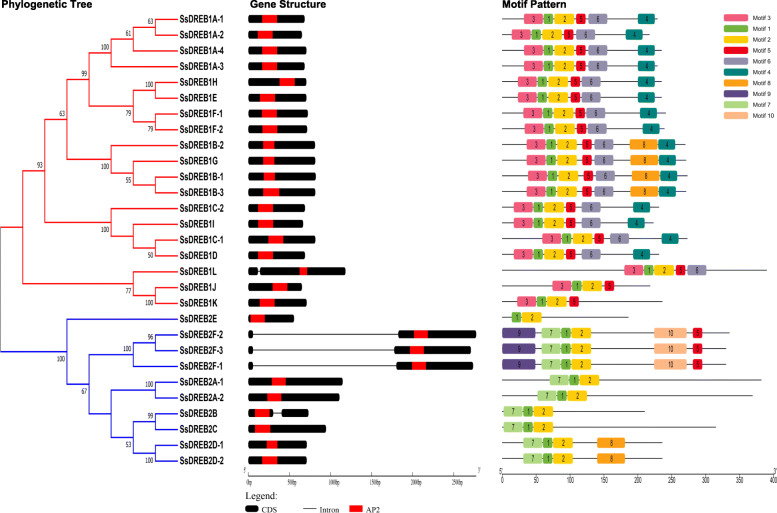
Phylogenetic relationships, gene structures and conserved protein motifs for the *SsDREB* genes. The phylogenetic tree was constructed based one the full-length protein sequences of 29 *SsDREB* alleles using MEGA 7.0. Exons and introns are represented by black boxes and lines, respectively. The AP2 domains are highlighted by red boxes. The numbers 1–10 of motifs are displayed in different color boxes

The exon-intron organizations and motifs of all *SsDREB* genes were examined in *S. spontaneum*. As shown in Fig. 3, all *SsDREB* genes had no intron except *SsDREB1L*, *SsDREB2F* and *SsDREB2B* with only one intron. The number and size of exon/intron among *SsDREB* alleles were highly conserved, while those in *SsDREB2F*, *SsDREB2F*-*2*’s intron were larger than other alleles. In addition, ten conserved motif sequences were detected (Fig. 3). All *SsDREB* genes contained Motif 1 and 2, which were related with AP2/ERF domain structure. Motif 3, 4 and 6 were only found in *DREB1* genes, whereas Motif 7 was unique to *DREB2* genes.

**Fig. 4:**
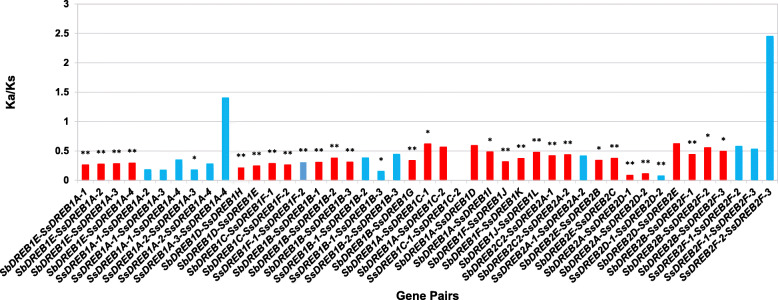
The Ka/Ks of *SsDREB* alleles and *SsDREB*-*SbDREB*. The blue boxes indicate the Ka/Ks of *SsDREB* allele genes, the red boxes indicate the Ka/Ks of orthologous between sorghum and *S. spontaneum.* The *p*-value < 0.05 is indicated by *. The *p*-value < 0.01 is indicated by **

To identify the evolutionary forces acting on the *SsDREB* genes with alleles, the ratio of the non-synonymous substitution rate to the synonymous substitution rate (Ka/Ks) was calculated. The Ka/Ks ratios between *SsDREB1A*-*3* and *SsDREB1A*-*4*, *SsDREB2F*-*2* and *SsDREB2F*-*3* were 1.401 and 2.450, respectively (Fig. 4), indicating that positive selection may be the dominant force driving the evolution of these two *SsDREB* genes.

### Expression analysis of *SsDREB* genes in *S. spontaneum*

The expression patterns of *SsDREB* genes in different tissues and developmental stages of *S. spontaneum* were investigated by using transcriptomic data. The RNA-seq results of *SsDREB1E*, *SsDREB1F*, *SsDREB1H* and *SsDREB2F* were corroborated by real time quantitative reverse transcription-PCR (qRT-PCR) in three tissues (the first, 6th and 15th segments of 11-day-old second leaves) of *S. spontaneum* (Additional File 4). There is a significant positive relationship (*R*^*2*^ = 0.7491) between the relative expression level and the Fragments Per transcript Kilobase per Million fragments mapped (FPKM) value (Additional File 4), supporting the reliability of the gene expression based on RNA-seq.

**Fig. 5:**
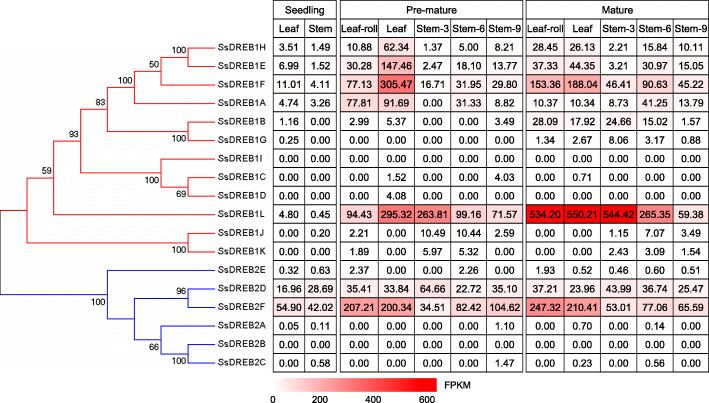
The expression pattern of *SsDREBs* based on FPKM in different tissues of different stages in *S. spontaneum*

**Fig. 6:**
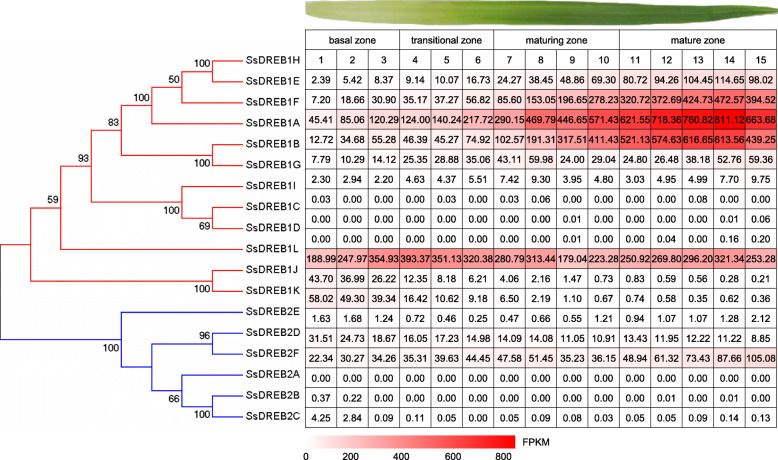
The expression pattern of *SsDREBs* based on FPKM across leaf gradients in *S. spontaneum*

**Fig. 7:**
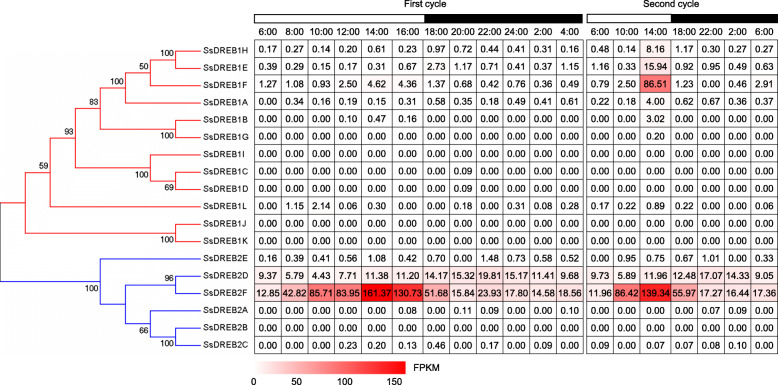
The expression pattern of *SsDREBs* based on FPKM at different time periods in leaves at *S. spontaneum*

Among the 18 *SsDREB* genes, 4 genes (*SsDREB1I*, *SsDREB2A*, *SsDREB2B* and *SsDREB2C*) were expressed at very low levels or undetectable in all examined tissues (Fig. 5). Transcripts of *SsDREB2D* was constitutively expressed in all these 12 tissues. The expression levels of *SsDREB1E*, *SsDREB1F*, *SsDREB1H* and *SsDREB2F* in leaves were higher than those in the stalks at different developmental stages. *SsDREB1A* exhibited much higher transcript levels in the leaves at maturing stage compared to other stages. The expression level of *SsDREB1L* increased with the maturity of the leaves, and gradually decreased from the top to bottom of the stem (Fig. 5). To further investigate the functions of *DREB* genes in the photosynthesis tissues of *S. spontaneum*. We exploited the continuously developmental gradient of the leaf to analyze the transcriptome of *SsDREBs*. Similarly to the maize [34], the leaf of *S. spontaneum* can be divided into four zones, including a basal zone (base, 1 cm above the leaf two ligule, sink tissue), a transitional zone (5 cm, 1 cm below the leaf one ligule, undergoing the sink-source transition), a maturing zone (10 cm, 4 cm above the leaf one ligule) and a mature zone (tip, 1 cm below the leaf two tip, fully differentiated and active C4 photosynthetic zones). Five genes (*SsDREB1C*, *SsDREB1D*, *SsDREB1I*, *SsDREB2A* and *SsDREB2B*) displayed undetectable or very low levels, suggesting that these genes play a very limited role in the developmental leaves in *S. spontaneum*. *SsDREB1A*, *SsDREB1E*, *SsDREB1F* and *SsDREB1H* showed higher expression levels in mature zone than those in other zones of the leaf, whereas *SsDREB1L* displayed higher expression levels in the transitional zone, *SsDREB1J* and *SsDREB1K* showed higher transcript levels in the basal zone (Fig. 6). For the *SsDREB2*-type genes, *SsDREB2F’s* transcript abundance gradually increased from the base to tip of the leaf, while the expression level of *SsDREB2D* gradually decreased from the base to tip of the leaf in *S. spontaneum* (Fig. 6). Additionally, we also collected samples for RNA-seq analysis at 2-h intervals over a 24-h period and 4-h intervals over an additional 24-h in *S. spontaneum*. *SsDREB2F* showed higher expression in the light period than that in the dark period over these two 24-h cycles, indicating this gene may play an important role in diurnal rhythms (Fig. 7).

**Fig. 8:**
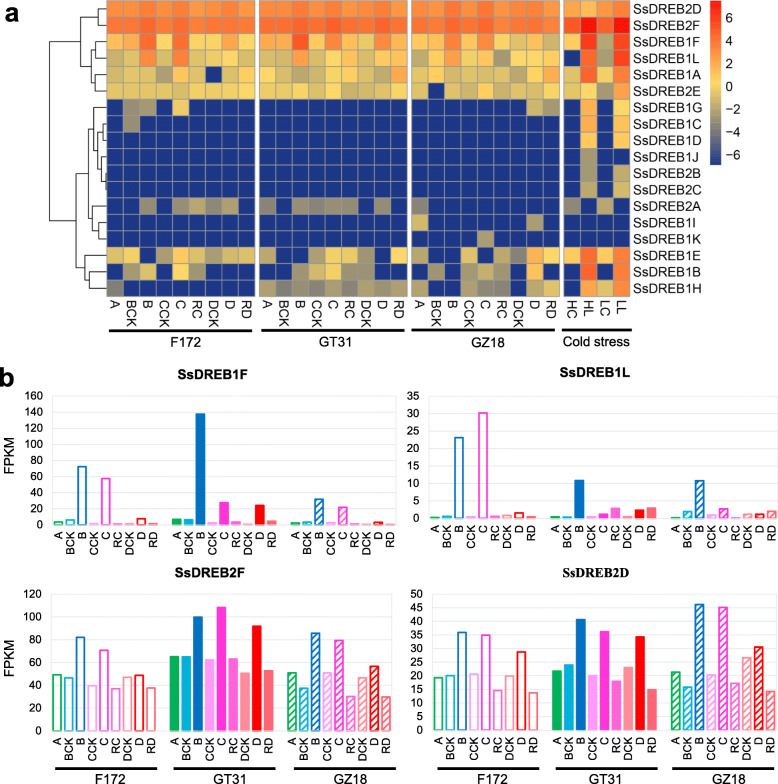
Expression profiles of 18 *SsDREB* genes under drought and cold stresses. **a** A heat map illustrating of gene expression levels of the 18 *SsDREB* genes under normal, drought and cold conditions. **b** The expression levels based on FPKM for *SsDREB1F*, *SsDREB1L*, *SsDREB2D*, and *SsDREB2F* under normal and drought conditions. F172, GT31, and GZ18 are three sugarcane cultivars. F172 is a strong drought tolerance cultivar, GT31 is a middle drought tolerance cultivar, and GZ18 is a drought sensitivity cultivar. A: the tissues under normal conditions; B: mild drought stress; BCK: the control group of mild drought stress; C: moderate drought stress; CCK: the control group of moderate drought stress; RC: re-watering after moderate drought stress treatment; D: severe drought stress; DCK: the control group of severe drought stress; RD: re-watering after severe drought stress treatment. HL, HC, LL and LC are *S. spontaneum* accessions with different polyploidy levels under cold stress treatment. HL: hyperploid clone 15–28 under cold stress; HC: hyperploid clone 15–28 under normal condition; LL: hypoploid clone 12–23 under cold stress; LC: hypoploid clone 12–23 under normal condition [35].

Furthermore, the transcriptome data of all *SsDREB* genes were analyzed in the primary meristem of the heart leaf in three drought-stressed sugarcane varieties. As illustrated in Fig. 8a, two *SsDREB1* genes and two *SsDREB2* genes were observed in response to drought stress, while the expression levels of *SsDREB1A* were slightly up-regulated after re-watering in three sugarcane varieties. *SsDREB1F* displayed similar expression patterns in these three sugarcane varieties, and its expression was gradually decreased with the increases of drought stress (Fig. 8b). What’s more, the greatest drought-inducible gene was found in *SsDREB1F* under the mild drought stress. The expression of *SsDREB1L* was up-regulated by dehydration in the drought-tolerant F172, which was also induced by the mild drought stress in other two varieties. Interestingly, the transcript abundances of *SsDREB1L* was increased slightly after re-watering under the moderate and severe drought stress conditions in GT31. Two *SsDREB2* genes, *SsDREB2D* and *SsDREB2F*, showed similar expression patterns with high expression levels. In contrast to the expressions under the normal growing conditions, the expressions of these two genes were up-regulated in response to dehydration, and then decreased after re-watering in all sugarcane varieties (Fig. 8b).

Finally, in order to investigate the response of *SsDREB* genes in cold stress, we analyzed the transcriptome expression profiles of all these genes in *S. spontaneum* under cold stress. Six *SsDREB* genes were induced by cold stress in hyperploid clone 15–28 (2n = 92) of *S. spontaneum*, and eight *SsDREB* genes were up-regulated in hypoploid clone 12–23 (2n = 54) (Fig. 8a). The greatest cold-inducible response was observed in *SsDREB1F*, whose expression was up-regulated more than 200-fold both in clone 15–28 and clone 12–23, in compare with expression under normal growing conditions. The induction response of *SsDREB1A*, *SsDREB1B*, *SsDREB1E* and *SsDREB1F* in clone 15–28 were higher than that in clone 12–23, while the expression levels of *SsDREB1L* and *SsDREB2F* in clone 12–23 were higher than that in clone 15–28 under cold stress. The expression of *SsDREB1H* and *SsDREB2D* were only up-regulated in response to cold stress in clone 12–23.

For the genes in tandemly duplicated regions, *SsDREB1F-2* and *SsDREB1H* showed higher expression levels in leaves than those in stalks at different developmental stages, moreover, the expression levels of *SsDREB1F-2*, *SsDREB1H* and *SsDREB1A-4* gradually increased from the base to tip of the leaf, whereas *SsDREB1B-3* and *SsDREB1G* displayed a lower levels in all tissues (Additional File 5). In addition, the expression of *SsDREB1F-2* was significantly up-regulated in response to dehydration in three sugarcane varieties, while other *SsDREB1* genes in tandemly duplicated clusters were expressed at very low levels or undetectable (Additional File 5).

## Discussion

The DREB-type transcription factors have been recently identified in many plants, for instance, *Arabidopsis* [1], *Brassica rapa* [36], rice [37, 38], barley [39], sorghum [40], and maize [9]. *DREB* genes also play a key role in plant response to multiple abiotic stresses [41]. Thus, it’s understandable that *DREB* genes may contribute to the enhanced stress tolerance and the improved production of sugarcane in field. However, the *DREB* genes have not been systematically studied in sugarcane because of its complex genetic background. In this study, 18 typical *DREB* genes in the *S. sponteaneum* genome were identified and analyzed using a bioinformatics approach to provide the clues for further functional investigations of *SsDREB* genes.

In the present study, 12 *SsDREB1* genes and six *SsDREB2* genes were identified in the *S. sponteaneum* genome, respectively. As a proximal species of sugarcane, there are ten *DREB1*-type genes and seven *DREB2*-type genes in the sorghum genome (Table 1). Phylogenetic analysis showed that a *DREB2*-type gene, *ABI4*, lost the orthologous gene in *S. sponteaneum*, which explains the reason why *S. sponteaneum* have one less *DREB2* genes, relative to the number of *DREB2* gene in sorghum. Previous research has reported that DREB1 family were expanded by the process of gene duplications [38]. Eleven tandemly duplicated *SsDREB1* allele genes were found in this paper, among them, the divergence times of *SsDREB1C*-*2* and *SsDREB1D* with their orthologous *SbDREB* are shorter than that of *S. spontaneum* and sorghum, suggesting that these two genes originated from tandem duplication after species differentiation between *S. spontaneum* and sorghum. Although all *SsDREB2* genes were derived from segmental duplication or whole-gnome duplication events, no gene expansion has occurred compared with sorghum. That is tandem duplication events, instead of segmental duplication or whole-gnome duplication events contributed to the expansion of *DREB* genes in *S. spontaneum*. Previous studies have been reported that duplications resulting in some gene families represented functional redundancy and/or divergence [42–44]. In this study, of the *SsDREB1* genes in Cluster 2 tandemly duplicated gene pairs, *SsDREB1F-2* and *SsDREB1H* have the same expression pattern at different developmental stages and leaf gradient segments (Additional File 5), whereas *SsDREB1B-3* and *SsDREB1G* expressed at very low levels, suggesting these tandemly duplicated genes may exist functional redundancy.

The V14 and E19 in the AP2/ERF domain are highly conserved and play a major role in the recognition and binding specificity of DRE *cis*-acting element [1]. However, some studies has reported that E19 is not conserved in DREB1 proteins in rice, wheat, barley and rye [41, 45]. Moreover, Dubouzet et al. reported that OsDREB1A do not has the glutamic acid in the E19 position, but binds specifically to the DRE element in the promoter of *rd29A* genes [46]. These results indicate that V14 may be more important than E19 for the recognition of DNA-binding sequence in DREB1 proteins. That is, deletion of this glutamic acid in the E19 position of *SsDREB1A*-*SsDREB1I* genes (Additional File 2) has little effect on their functions. TFs only function in the nucleus, the regulation of their entry into nucleus is significant for their function. The NLS medicates the entry of TFs, and the TFs without NLS enters the nucleus by the interaction with the TFs with NLS [45]. The DREB1-type transcription factors are distinctly different from other subgroup DREBs, they have a NLS sequence ‘PKK/RPAGRxKFxETRHP’ at the upstream of AP2/ERF domain, while DREB2 proteins possess a PKK-like NLS sequence ‘RKxPAKGSKKGCMxGKGGPENxx’ immediately upstream of the AP2/ERF domain [47, 48]. In the current study, all SsDREB1 proteins had a NLS sequence ‘P/KKR/KP/RA/TGRxKFRETRHP’, and most SsDREB2 proteins possessed a PKK-like NLS, except for SsDREB2E (Additional File 2), indicating that *SsDREB2E* might function differently from other *SsDREB2* genes.

DREBs are considered as the master regulators of various abiotic stress responses, and also involved in the developmental processes of plants. For instance, *OsDREB2A*, *OsDREB2B*, and *OsAIB4* were reported to involve in the embryo and endosperm development in rice [49]. In *Arabidopsis*, *ABI4* was involved in the seed development and the lateral root formation [50, 51]. Given gene expression patterns are highly corrected with their functions in plants [52], *SsDREB1E*, *SsDREB1F*, *SsDREB1H*, and *SsDREB2F* displayed higher expression levels in leaves than culms at different developmental stages of *S. spontaneum* in the present study (Fig. 5), suggesting these four genes might play a major role in the leaf development throughout the plant life cycle, while *SsDREB1A* showed related to the leaf development only at maturing stage. And the gradually increased expression pattern from the near-terrestrial end to the distal of steam at the stage of sugar accumulating suggested that *SsDREB1L* may play role relative to sugar metabolism. Constitutively expression of *SsDREB2D* in all tissues indicated that this gene may has a central role throughout *S. spontaneum* life cycle.

Li et al. reported that in the region of transition from the sink to source tissues of the leaf, the transcript abundance of genes associated with the photosynthetic machinery is increased [34], *SsDREB1L* exhibited higher expression levels in this transitional zone than other regions (Fig. 6), suggesting that this gene could be associated with the photosynthetic machinery of *S. spontaneum*. The peak expression levels of *SsDREB1J*, *SsDREB1K* and *SsDREB2D* in the undifferentiated basal region suggested that these three gene may regulate the basic cellular activities. The distal region of the leaf was fully differentiated with highest levels of photosynthesis, thus, the dominant expression levels of *SsDREB1A*, *SsDREB1E*, *SsDREB1F*, *SsDREB1H* and *SsDREB2F* in the distal region of the leaf indicated that these five genes may be highly correlated with the photosynthetic reactions in leaves. Previous researches have documented that the expression levels of some plant genes are effected by circadian rhythm, which gives plants the innate ability to measure time, and allows them to anticipate daily changes in the environment and to coordinate the developmental and metabolic processes induced by the environmental factors [53–58]. However, only *SsDREB2F* showed varied expression in the mature leaf during the diurnal cycle (Fig. 7), indicating that this gene might regulate the photosynthesis in mature sugarcane.

Previous efforts have been made to demonstrate that *DREB1s* or *DREB2s* involve the process regulating the stress in a number of plants, including maize, rice, cotton, and *S. miltiorrhiza* [19, 25, 26, 46, 59]. In this work, expression profile analysis revealed that *SsDREB1F*, *SsDREB1L*, *SsDREB2D*, and *SsDREB2F* were induced by drought stress in three sugarcane varieties (Fig. 8), suggesting these four *DREB* genes might play a key role in response to dehydration in sugarcane. The expression level of *SsDREB1L* was increased after re-watering in GT31, a sugarcane variety which is not drought-tolerant with the good recovering ability. This result indicated that this gene might help plant recover from drought stress in sugarcane. In addition, the expression levels of *SsDREB1A*, *SsDREB1B*, *SsDREB1E*, *SsDREB1F*, *SsDREB1H*, *SsDREB1L*, *SsDREB2D* and *SsDREB2F* were up-regulated under cold stress in *S. spontaneum*, indicating that these eight genes may be involved in responding to cold stress in *S. spontaneum*.

## Conclusions

In summary, the present study identified 12 *DREB1* genes and 6 *DREB2* genes from *S. spontaneum*, and renamed them as *SsDREB1A*-*SsDREB1L*, and *SsDREB2A*-*SsDREB2F* on the basis of their chromosomal locations. Phylogenetic analysis based on the orthologous from sorghum, rice, maize, and *Arabidopsis* revealed that a *DREB2*-type gene, *ABI4*, was lost during the evolution of *S. spontaneum*. Analysis of gene duplication showed that tandem duplication events contributed to the expansion of *DREB1*-type gene in *S. spontaneum*. In addition, these genes showed functional role in the sugarcane growth and development, photosynthesis, dehydration and cold stress response. However, how these *DREB* genes participate in the processes of development, and stress response remains to be further elucidated. Our present findings offer a useful information to understand the physiological functions of *SsDREBs* in sugarcane.

## Materials and methods

### Plant materials

The founding *Saccharum* species, *S. spontaneum* SES208 (originated in USA) was used in this paper [60]. The plant material was identified by Irvine JE [61], and the *Saccharum* species was planted in the campus of Fujian Agricultural and Forestry University (Fuzhou, China). The collection of *S. spontaneum* and the performance of experimental research on such plant were complied with the national guidelines of China.

The tissues of *S. spontaneum* at different developmental stages were obtained from leaf roll, leaf, top immature internode (Stem-3), premature internode (Stem-9) and mature internode (Stem-15) as previously described [62–64]. For leaf gradient experiment, *S. spontaneum* plants were grown under the following conditions: light intensity of 350 μmol/m^2^/s, 14:10 L/D, 30 °C L/22 °C D and 60% relative humidity. 11-day-old second leaves were collected after planting 3 h into the light period and were cut into 15 1-cm segments followed the approach described by Li et al. [34]. The mature leaves of *S. spontaneum* for the diurnal cycle experiment were collected at 12 time points (6 a.m., 8 a.m., 10 a.m., noon, 2 p.m., 4 p.m., 6 p.m., 8 p.m., 10 p.m., midnight, 2 a.m., 4 a.m.) and 7 time points (6 a.m., 10 a.m., 2 p.m., 6 p.m., 10 p.m., 2 a.m., 6 a.m.) from March 2 to 3, 2017 [64, 65].

The transcriptome data of sugarcane with drought treatment were obtained from Sugarcane Research Institute of Guangxi Academy of Agricultural Sciences (Nanning, China). We collected the first leaves of three 7-month-old sugarcane cultivars (F172, strong drought tolerance; GT31, middle drought tolerance; GZ18, drought sensitivity) under normal conditions, mild drought stress, moderate drought stress, severe drought stress and re-watering for RNA-seq library construction. The transcriptome data of *S. spontaneum* under cold stress were obtained from Yang et al [35].

### Identifications of *SsDREB* genes

A Hidden Markov Model (HMM) profile of the AP2/ERF domain (PF00847) was obtained from the Pfam protein family database (http://pfam.xfam.org/) [66] and used to identify the proteins which contain AP2/ERF domain(s). Then we obtained *DREB* genes based on the similarities of the amino acid sequence and the number of AP2/ERF domains. Finally, the physical and chemical properties including AA, MW, theoretical pI, GRAVY, AI, and II of putative SsDREB proteins were calculated by the online ExPASy-ProtParam tool (http://web.expasy.org/protparam/). Manual annotation was performed for the genes that were incorrectly predicted. Additionally, *DREB* orthologous genes from sorghum, rice, maize, and *Arabidopsis* were collected [9, 37, 38, 40, 67].

### Sequence analysis

The AP2/ERF domain sequences of identified 29 SsDREB proteins were included in multiple sequence alignments using DNAMAN with default parameters. The exon-intron organization of *SsDREB* genes was determined based on their coding sequence alignments and respective genomics sequences using the online program Gene Structure Display Server (GSDS: http://gsds.gao-lab.org/) [68]. Conserved motif in SsDREB proteins were predicted using TBtools software with number of motifs to find: 10 and minimum-maximum width to find: 6–50 [69]. The non-synonymous (Ka) and synonymous (Ks) substitution ratios were calculated by the easy_KaKs calculation program [70]. The divergence time (T) was calculated by T = Ks/ (2 × 6.1 × 10^− 9^) × 10^− 6^ Mya [71].

### Phylogenetic analysis

The sequences of DREB proteins were aligned using MUSCLE in MEGA (version 7.0) with default parameters [72]. A phylogenetic tree based on the alignment was constructed using MEGA (version 7.0) with the neighbor-joining (NJ) method with the bootstrap test replicated 1000 times, the Poisson model, and Pairwise deletion [73, 74]. The result was imported into the Interactive Tree Of Life (iTOL) program to create the phylogenetic tree [75].

### Chromosomal distribution and gene duplication

The physical location of *SsDREBs* on the chromosomes was obtained from the database of *S. spontaneum* genome. MapInspect software (http://www.softsea.com/download/MapInspect.html) was employed to visualize the chromosomal distribution of deduced *SsDREB* genes according to their initial position and length of chromosome. To analyze the duplication pattern for each *SsDREB* gene, the BLASTP program (E-value < 10^− 5^) and Multiple Collinearity Scan toolkit (MCScanX) were used [76].

### Expression profiling analysis of *DREBs* in *S. spontaneum* based on RNA-seq

HiSeq™ 2500 platform (Illumina Inc., CA, USA) by the Novogene Bioinformatics Technology Co., Ltd. (Beijing, China). The reads obtained from the sequencing instruments under drought and cold stress were filtered to remove adapters and low-quality reads by Trimomatic [77]. The reference genome of *S. spontaneum* AP85–441 (v20180123) is constructed to be indexed for further analysis by the align_and_estimate_abundance.pl of Trinity (version 2.8.5) [78]. Transcript expression levels of individual genes were quantified using FPKM values (fragments per kilobase of exon per million fragments mapped) by align_and_estimate_abundance.pl in Trinity [78] and value of the gene was calculated using the RNA-Seq by the Expectation-Maximization (RSEM) method.

### Experimental validation of *DREB* gene expression level by qRT-PCR

RNA of each sample was in reverse transcription with the StarScript II First-strand cDNA Synthesis Mix with gDNA Remover (GenStar, A224–10) following the manufacturer’s instructions. The qRT-PCR amplification was carried out using 2 × RealStar Green Fast Mixture (GenStar, A301–10) on a Multicolor Real-Time PCR Detection System (Bio-Rad) and the reaction program was refer to the two steps method of the protocol from this kit: 95 °C for 2 min, 40 cycles of 95 °C for 15 s and 60 °C for 30 s, The expression of glyceraldehyde-3-phosphate dehydrogenase gene (*GAPDH*) and Eukaryotic elongation factor 1a (*eEF-1a*) were used as internal control [79] and the primers of *DREB* genes are listed in Additional File 6.

## Supplementary Information

**Additional file 1 **Sequence features of DREBs in *S. spontaneum*.

**Additional file 2.** Alignment of the deducted protein sequences of SsDREBs. The rectangle in black and red represent AP2/ERF domain and motifs (DASW, LWSY, CMIV-1), respectively, the black line represent the NLS sequence.

**Additional file 3 **The segmental or whole-genome duplicated *DREB* genes in *S.spontanuem*.

**Additional file 4 **qRT-PCR verification of *SsDREB* genes in *S. spontanenum*. **a** Comparison of qRT-PCR and RNA-seq data of *SsDREB* genes. **b** Correlation coefficient between RNA-seq (X axis) and qRT-PCR (Y axis) of four *SsDREB* genes.

**Additional file 5 **Expression pattern of *SsDREB* genes in tandemly duplicated regions in *S. spontaneum*.

**Additional file 6.** Gene primers used for qRT-PCR analysis.

## Data Availability

All sequence data and phylogeny data during the current study were deposited in the Treebase repository (http://purl.org/phylo/treebase/phylows/study/TB2:S28119). The genomic data of *S. spontaneum* was generated in our own laboratory [80] (accession numbers in Genbank: QVOL00000000).

## Abbreviations

TFs: Transcription factors
DREBs: Dehydration responsive element binding proteins
ABA: Abscisic acid
AP2/ERFBP: APETALA2/ethylene-responsive element-binding protein
AA: The length of protein sequences
MW: The molecular weight
pI: The theoretical isoelectric point
AI: The aliphatic index
GRAVY: The grand average of hydropathicity
II: The instability index
NLS: Nuclear localization signal
Ka: Non-synonymous
Ks: Synonymous
Mya: Million years ago
qRT-PCR: Real time quantitative reverse transcription-polymerase chain reaction
FPKM: Fragments Per transcript Kilobase per Million fragments mapped
RNA-seq: RNA Sequencing
HMM: Hidden Markov Model
NJ: Neighbor-joining
RSEM: RNA-Seq by the Expectation-Maximization
GAPDH: Glyceraldehyde-3-phosphate dehydrogenase
eEF-1a: Eukaryotic elongation factor 1a
