# Supplementary material for: Genome-wide identification and expression profiling of DREB genes in Saccharum spontaneum
Source: BMC Genomics. 2021 Jun 17;22:456. doi: 10.1186/s12864-021-07799-5 (PMC8212459; doi:10.1186/s12864-021-07799-5)
Supplement: Supplementary file 1 — Additional file 1 Sequence features of DREBs in S. spontaneum. [file 12864_2021_7799_MOESM1_ESM.docx]

**Additional File 1:** Sequence features of DREBs in *S. spontaneum*.

| Gene ID | Gene name | Chr | Start site | End site | AA | MW | PI | II | AI | GRVY |
| --- | --- | --- | --- | --- | --- | --- | --- | --- | --- | --- |
| Sspon.002A0012760 | SsDREB1A-1 | Chr2A | 27398387 | 27399073 | 229 | 24356.94 | 4.99 | 77.01 | 59.43 | -0.385 |
| Sspon.002B0011090 | SsDREB1A-2 | Chr2B | 26539956 | 26540610 | 217 | 23298.73 | 4.95 | 78.35 | 63.13 | -0.353 |
| Sspon.002C0014070 | SsDREB1A-3 | Chr2C | 30543015 | 30543701 | 229 | 24383.02 | 4.99 | 76.17 | 61.14 | -0.365 |
| Sspon.002D0010170 | SsDREB1A-4 | Chr2D | 22126308 | 22127015 | 235 | 25173.89 | 4.99 | 75.27 | 61.19 | -0.373 |
| Sspon.002A0012780 | SsDREB1B-1 | Chr2A | 27420092 | 27420913 | 273 | 28270.46 | 6.53 | 65.04 | 66.34 | -0.29 |
| Sspon.002B0011120 | SsDREB1B-2 | Chr2B | 26564597 | 26565409 | 270 | 28084.39 | 8.84 | 62.17 | 66.7 | -0.293 |
| Sspon.002D0010130 | SsDREB1B-3 | Chr2D | 22092288 | 22093103 | 271 | 28115.35 | 6.44 | 65.05 | 67.53 | -0.279 |
| Sspon.002B0011140 | SsDREB1C-1 | Chr2B | 26597272 | 26598093 | 273 | 29564.97 | 5.16 | 53.52 | 69.52 | -0.404 |
| Sspon.002D0010210 | SsDREB1C-2 | Chr2D | 22208944 | 22209639 | 231 | 24954.69 | 4.95 | 55.04 | 70.74 | -0.419 |
| Sspon.002B0011150 | SsDREB1D | Chr2B | 26612975 | 26613670 | 231 | 24962.75 | 4.95 | 55.15 | 71.17 | -0.403 |
| Sspon.002C0014080 | SsDREB1E | Chr2C | 30554216 | 30554923 | 235 | 25149.95 | 4.95 | 67.62 | 63.23 | -0.291 |
| Sspon.002C0014100 | SsDREB1F-1 | Chr2C | 30628112 | 30628837 | 241 | 25675.22 | 4.94 | 63.09 | 57.72 | -0.376 |
| Sspon.002D0010150 | SsDREB1F-2 | Chr2D | 22113187 | 22113903 | 239 | 25285.76 | 4.82 | 70.15 | 56.99 | -0.415 |
| Sspon.002D0010140 | SsDREB1G | Chr2D | 22107221 | 22108036 | 271 | 28213.41 | 6.22 | 64.97 | 67.53 | -0.296 |
| Sspon.002D0010160 | SsDREB1H | Chr2D | 22117638 | 22118345 | 235 | 25128.89 | 4.95 | 63.88 | 64.09 | -0.271 |
| Sspon.002D0010220 | SsDREB1I | Chr2D | 22226936 | 22227607 | 223 | 24010.63 | 4.78 | 48.8 | 70.22 | -0.4 |
| Sspon.003C0002010 | SsDREB1J | Chr3C | 4000829 | 4001482 | 218 | 23398.4 | 10.53 | 61.96 | 59.82 | -0.544 |
| Sspon.003C0002641 | SsDREB1K | Chr3C | 5228786 | 5229496 | 236 | 25328.31 | 6.23 | 61.22 | 60.17 | -0.519 |
| Sspon.008B0021400 | SsDREB1L | Chr8B | 60925087 | 60926302 | 390 | 41745.7 | 8.1 | 62.62 | 67.77 | -0.473 |
| Sspon.001A0011140 | SsDREB2A-1 | Chr1A | 26433521 | 26434669 | 382 | 41131.85 | 7.01 | 60.56 | 47.7 | -0.597 |
| Sspon.001B0035400 | SsDREB2A-2 | Chr1B | 94333831 | 94334940 | 369 | 39629.96 | 6.98 | 60.21 | 46.75 | -0.618 |
| Sspon.001C0004630 | SsDREB2B | Chr1C | 10628954 | 10629687 | 210 | 22471.37 | 8.62 | 52.33 | 65.81 | -0.523 |
| Sspon.001D0004300 | SsDREB2C | Chr1D | 9931730 | 9932677 | 315 | 33368.46 | 6.17 | 43.45 | 70.89 | -0.351 |
| Sspon.003B0031080 | SsDREB2D-1 | Chr3B | 88084363 | 88085075 | 236 | 26145.04 | 5.3 | 43.15 | 66.53 | -0.686 |
| Sspon.003C0034300 | SsDREB2D-2 | Chr3C | 84081158 | 84081868 | 236 | 26143.06 | 5.3 | 45.91 | 66.95 | -0.67 |
| Sspon.007A0007600 | SsDREB2E | Chr7A | 15178438 | 15179068 | 186 | 20362.44 | 5.55 | 60.66 | 56.29 | -0.6 |
| Sspon.007A0013130 | SsDREB2F-1 | Chr7A | 30666109 | 30668933 | 330 | 35503.44 | 5.09 | 50.01 | 54.85 | -0.693 |
| Sspon.007C0011560 | SsDREB2F-2 | Chr7C | 26785987 | 26788979 | 335 | 35945.79 | 5 | 50.77 | 52.87 | -0.68 |
| Sspon.007D0011170 | SsDREB2F-3 | Chr7D | 26811739 | 26814577 | 330 | 35515.36 | 5.08 | 51.18 | 53.36 | -0.719 |

Note: Chr, chromosome numbers; AA, the length of protein sequences; MW, molecular weight; PI, isoelectric point; II, instability index; AI, Aliphatic index; GRVY, grand average of hydropathicity.
