# Supplementary material for: Genome-wide identification and expression profiling of DREB genes in Saccharum spontaneum
Source: BMC Genomics. 2021 Jun 17;22:456. doi: 10.1186/s12864-021-07799-5 (PMC8212459; doi:10.1186/s12864-021-07799-5)
Supplement: Supplementary file 2 — Additional file 2. Alignment of the deducted protein sequences of SsDREBs. The rectangle in black and red represent AP2/ERF domain and motifs (DASW, LWSY, CMIV-1), respectively, the black line represent the NLS sequence. [file 12864_2021_7799_MOESM2_ESM.pdf]

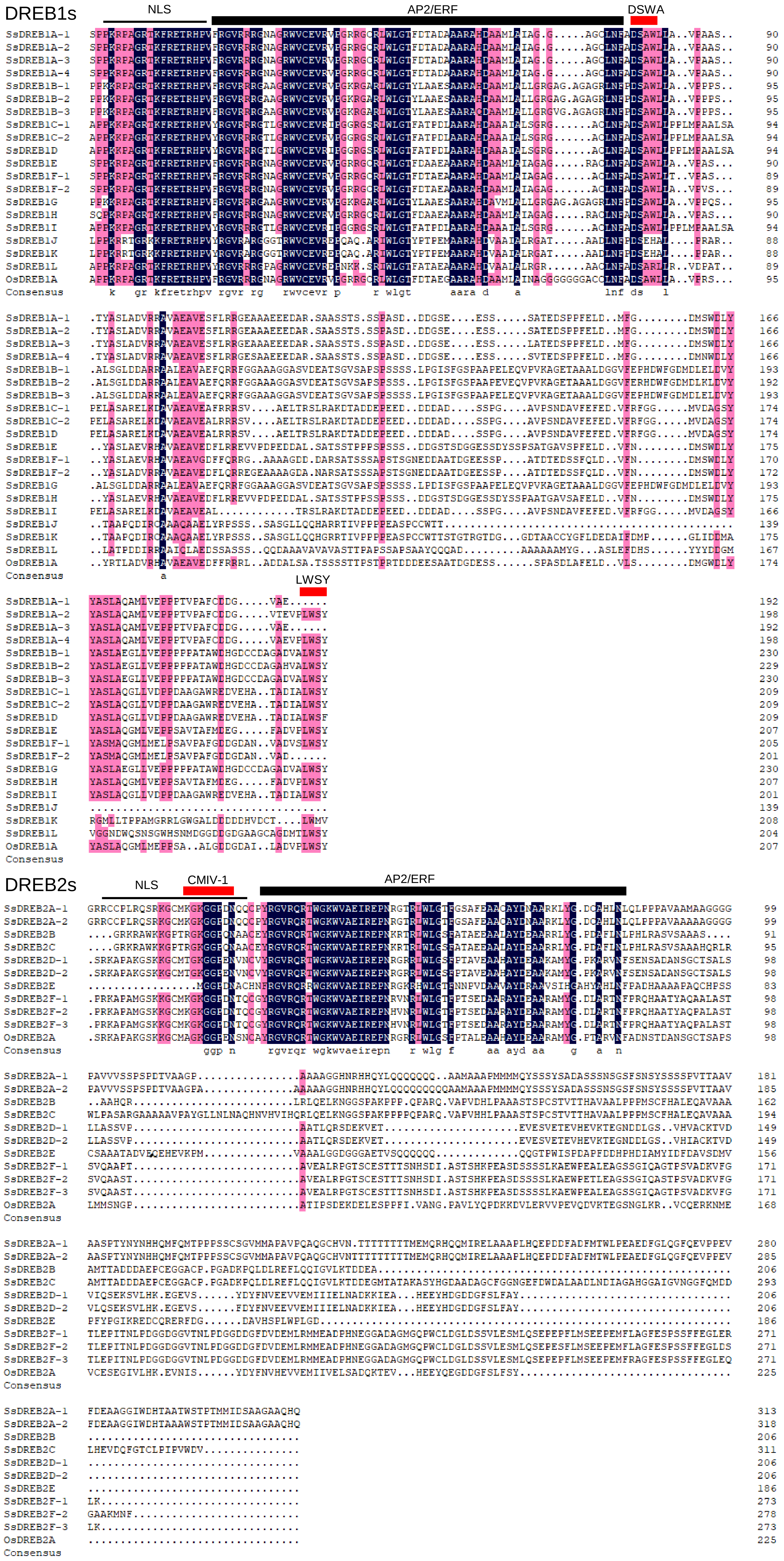
DREB1s
NLS
AP2/ERF
DSWA
LWSY
DREB2s
NLS
CMIV-1
AP2/ERF
