# Supplementary material for: Genome-wide identification and expression profiling of DREB genes in Saccharum spontaneum
Source: BMC Genomics. 2021 Jun 17;22:456. doi: 10.1186/s12864-021-07799-5 (PMC8212459; doi:10.1186/s12864-021-07799-5)
Supplement: Supplementary file 3 — Additional file 3 The segmental or whole-genome duplicated DREB genes in S.spontanuem. [file 12864_2021_7799_MOESM3_ESM.docx]

Additional File 3 The segmental duplicated *DREB* genes in *S.spontanuem*.

| Gene ID | Gene name | Chromosome | Start site | End site |
| --- | --- | --- | --- | --- |
| Sspon.002A0012760 | SsDREB1A-1 | Chr2A | 27398387 | 27399073 |
| Sspon.002C0014070 | SsDREB1A-3 | Chr2C | 30543015 | 30543701 |
| Sspon.002C0014100 | SsDREB1F-1 | Chr2C | 30628112 | 30628837 |
| Sspon.002D0010150 | SsDREB1F-2 | Chr2D | 22113187 | 22113903 |
| Sspon.002D0010130 | SsDREB1B-3 | Chr2D | 22092288 | 22093103 |
| Sspon.003C0002010 | SsDREB1J | Chr3C | 4000829 | 4001482 |
| Sspon.008B0021400 | SsDREB1L | Chr8B | 60925087 | 60926302 |
| Sspon.001A0011140 | SsDREB2A-1 | Chr1A | 26433521 | 26434669 |
| Sspon.001B0035400 | SsDREB2A-2 | Chr1B | 94333831 | 94334940 |

| Sspon.001C0004630 | SsDREB2B | Chr1C | 10628954 | 10629687 |
| --- | --- | --- | --- | --- |
| Sspon.001D0004300 | SsDREB2C | Chr1D | 9931730 | 9932677 |
| Sspon.003B0031080 | SsDREB2D-1 | Chr3B | 88084363 | 88085075 |
| Sspon.003C0034300 | SsDREB2D-2 | Chr3C | 84081158 | 84081868 |
| Sspon.007A0007600 | SsDREB2E | Chr7A | 15178438 | 15179068 |
| Sspon.007A0013130 | SsDREB2F-1 | Chr7A | 30666109 | 30668933 |
| Sspon.007C0011560 | SsDREB2F-2 | Chr7C | 26785987 | 26788979 |
| Sspon.007D0011170 | SsDREB2F-3 | Chr7D | 26811739 | 26814577 |
