# Supplementary figures and images for: Genome-wide identification and expression profiling of DREB genes in Saccharum spontaneum
Source: BMC Genomics. 2021 Jun 17;22:456. doi: 10.1186/s12864-021-07799-5 (PMC8212459; doi:10.1186/s12864-021-07799-5)

**a****SsDREB1E**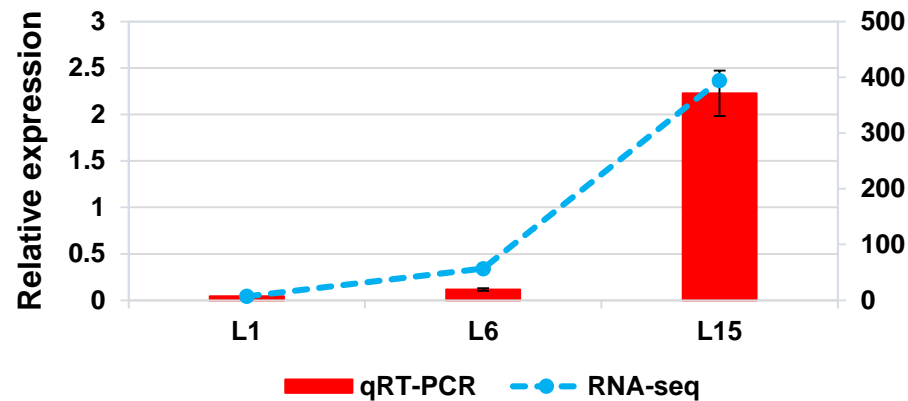**SsDREB1F**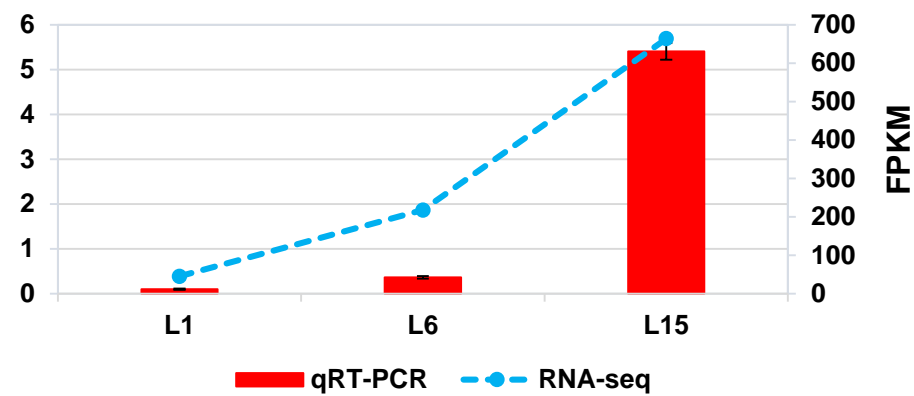**SsDREB1H**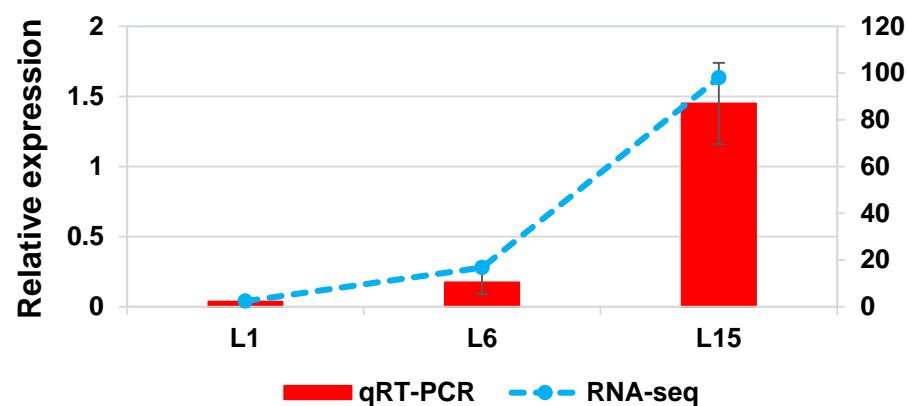**SsDREB2F**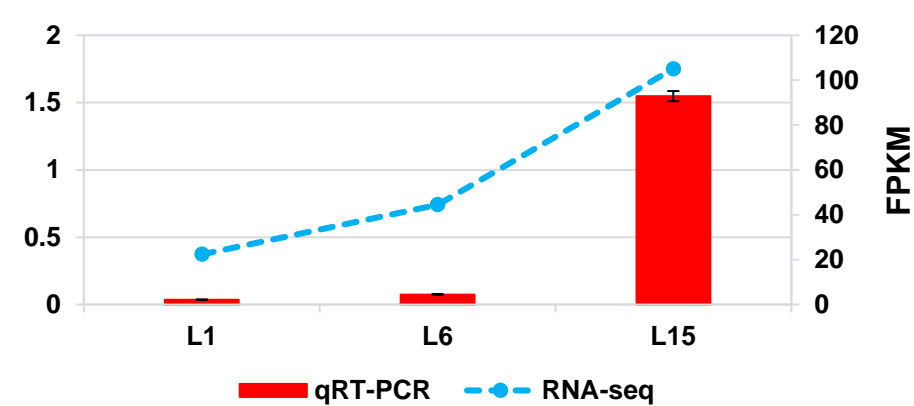**b**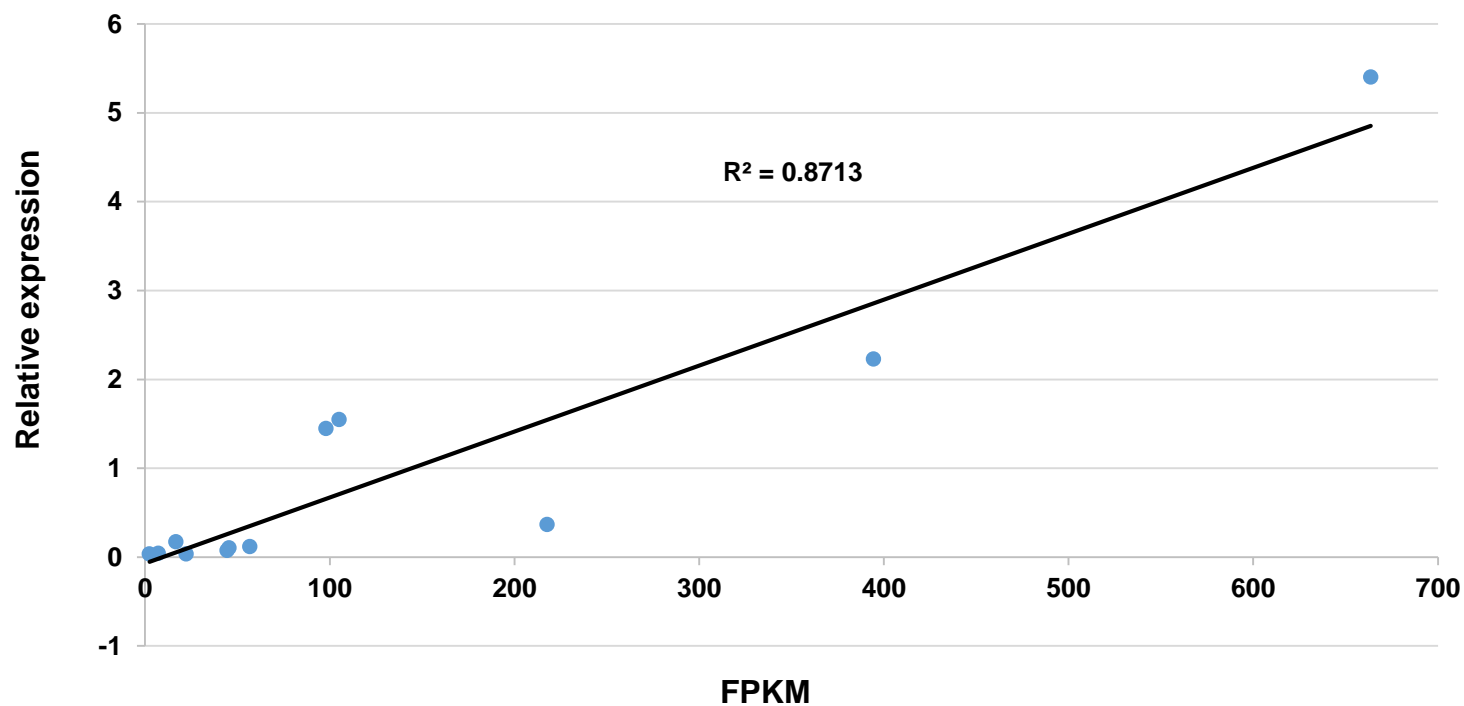

Supplement: Supplementary file 4 — Additional file 4 qRT-PCR verification of SsDREB genes in S. spontanenum. a Comparison of qRT-PCR and RNA-seq data of SsDREB genes. b Correlation coefficient between RNA-seq (X axis) and qRT-PCR (Y axis) of four SsDREB genes. [file 12864_2021_7799_MOESM4_ESM.pdf]
