# Supplementary material for: Genome-wide identification and expression profiling of DREB genes in Saccharum spontaneum
Source: BMC Genomics. 2021 Jun 17;22:456. doi: 10.1186/s12864-021-07799-5 (PMC8212459; doi:10.1186/s12864-021-07799-5)
Supplement: Supplementary file 6 — Additional file 6. Gene primers used for qRT-PCR analysis. [file 12864_2021_7799_MOESM6_ESM.docx]

**Additional File 6:** Gene primers used for qRT-PCR analysis.

| Gene ID | Forward Primer | Reverse Primer |
| --- | --- | --- |
| *SsDREB1E* | GGGAGTCCTCTGATTATTCCTCT | GTAGTACAGGTCCCAGCTCAT |
| *SsDREB1F* | CAATGACATGAGCTGGGATCT | GTAGCTCCACAGTGACACATC |
| *SsDREB1H* | GGGAGTCCTCTGATTATTCCTCT | GTAGTACAGGTCCCAGCTCAT |
| *SsDREB2F* | GACGCTGGAACCTATCACAAA | CCATCATCCTCAGCATCTCATC |
